# Differential role of prefrontal, temporal and parietal cortices in verbal and figural fluency: Implications for the supramodal contribution of executive functions

**DOI:** 10.1038/s41598-019-40273-7

**Published:** 2019-03-06

**Authors:** Elham Ghanavati, Mohammad Ali Salehinejad, Vahid Nejati, Michael A. Nitsche

**Affiliations:** 1 Department of Psychology, Islamic Azad University, Science & Research Branch, Tehran, Iran; 2 Department of Psychology and Neurosciences, Leibniz Research Centre for Working Environment and Human Factors, Dortmund, Germany; 3 Institute for Cognitive and Brain Sciences, Shahid Beheshti University, Tehran, Iran; 4 International Graduate School of Neuroscience, Ruhr University Bochum, Bochum, Germany; 5 Faculty of Psychology and Educational Sciences, Department of Psychology, Shahid Beheshti University, Tehran, Iran; 6 Department of Psychology, University of Regensburg, Regensburg, Germany; 7 Department of Neurology, University Medical Hospital Bergmannsheil, Bochum, Germany

## Abstract

Verbal and figural fluency are related to executive functions (EFs), but the extent to which they benefit from executive resources and their respective cortical representations is not clear. Moreover, different brain areas and cognitive functions are involved in fluency processing. This study investigated effects of modulation of cortical excitability in the left dorsolateral prefrontal cortex (l-DLPFC), left temporal area and right posterior parietal cortex (r-PPC) with transcranial direct current stimulation (tDCS), on verbal and figural fluency. Fifteen healthy adult participants received anodal l-DLPFC (F3), anodal left temporal (T3), anodal r-PPC (P4) and sham tDCS (15 min, 1.5 mA). After five minutes of stimulation, participants underwent the verbal fluency (i.e., semantic and phonemic fluency tasks) and figural fluency tasks. Participants significantly generated more words with phonemic cues during anodal l-DLPFC tDCS and more words with semantic cues during both anodal left temporal and anodal l-DLPFC tDCS. In contrast, they generated more unique figures under anodal r-PPC and anodal l-DLPFC tDCS. Our results implicate that prefrontal regions and EFs are shared anatomical correlates and cognitive processes relevant for both, verbal and figural fluency (supramodal contribution of DLPFC activation), whereas r-PPC and left temporal cortex are more specifically involved in figural and semantic fluency (modality-specific contribution).

## Introduction

Fluency is defined as the ability to maximize unique response productions, and at the same time to avoid or minimize response repetition^1^. It is generally divided into verbal and nonverbal categories. While verbal fluency is referred to the ability to generate and express words compatible with required criteria^2^, nonverbal fluency involves generation of unique, novel and original nonverbal responses, which is usually measured by figural or design fluency^3^. Fluency tasks - especially in the verbal modality - are frequently used to test executive functioning and some studies suggest that all forms of fluency, independent from modality, represent general executive functions (EFs)^4^. In contrast, recent evidence suggests distinct anatomical and cognitive correlates of semantic and phonemic fluency, two major aspects of verbal fluency^5,6^. Moreover, figural fluency, a nonverbal analogue of verbal fluency^7^, is related not only to EF but also to visuomotor abilities. That said, it is not well-studied how verbal and figural fluency are interrelated, whether there are shared and distinct brain regions and cognitive functions underlying them and to what extent the EFs contribute to verbal/figural fluency.

Previous studies show that fluency modalities, including verbal and nonverbal fluency (i.e., figural fluency, design fluency), are associated with executive functioning^4,8–10^. Fluency tests are regarded as major measures of EFs and frontal lobe functions. Some studies, however, suggest that these tests should not be used as “frontal lobe tests” per se, but rather as tests of specific executive functioning^9^. Verbal fluency tasks (i.e., semantic and phonemic fluency) require attention, semantic retrieval, processing speed and working memory all of which are related to EFs^5,11,12^. Similarly, figural fluency performance is a goal-directed action requiring executive functioning^13^, inhibition^14^, visuospatial functions^15^, updating and mental flexibility^14,16–18^. In this line findings from neuroimaging and lesion studies have shown the involvement of prefrontal areas and specifically, the dorsolateral prefrontal cortex (DLPFC), in fluency performance^3,19–21^. Converging evidence from clinical populations confirm reduced activity in the left dorsolateral prefrontal cortex (DLPFC) related to impaired verbal fluency ability independent of modality^22–24^. However, when it comes to fluency within specific modalities (i.e., semantic fluency, phonemic fluency, figural fluency), other cognitive processes and their associated brain areas are involved.

Recent studies indicate that verbal and figural fluency may be processed at least partially by distinct brain regions and cognitive functions^5,6^. Evidence from lesion studies shows that left hemispheric lesions produce more severe deficits in verbal fluency than right-sided lesions, whereas figural fluency is particularly sensitive to right-sided brain lesions^25^. Furthermore, distinct brain regions are shown to be involved in verbal fluency depending on task specifics^26^. For example, neuroimaging studies found greater activation in the left hemisphere during semantic and phonemic fluency tasks especially in the anterior cingulate and left prefrontal regions^19^, whereas the medial prefrontal cortex (mPFC) and medial temporal regions are specifically relevant for semantic fluency^5,27^. These findings suggest that the DLPFC might have a supramodal contribution to fluency similar to its well-documented role in EFs^28^, whereas other brain areas including the mPFC and medial temporal region contribute to specific verbal fluency functions.

Similarly, figural fluency performance seems to be dependent on different brain areas with general and specific contributions. Figural fluency tests require participants to generate unique line drawings across five dots while trying to avoid repetitions, which is an example of motor planning and goal-directed action. It requires cognitive control to maintain information and coordinate action sequences which is related to DLPFC activation^28,29^. Findings from patients with frontal lobe lesions confirm this by demonstrating impaired performance in figural fluency tests^30^. On the other hand, figural fluency depends also on visual-spatial functions^15^. Parietal regions, especially the posterior parietal cortex (PPC), are involved in visuospatial attention^31^, visually guided motor planning^32^, spatial representation and updating and retrospective coding of visual space (which is specifically important in figural fluency performance), that have a crucial role in transforming sensory input into motor output and guiding action^16–18^. Similar to the PFC, the PPC is significantly involved in integrating sensory information, specifically visuospatial information, in favour of goal-directed movements^33,34^ and contributes to higher cognitive functions such as attention and intention^33,34^. Specifically, imaging findings show that the PFC and PPC interact in achieving cognitive control in a way that neural activity in the prefrontal areas precedes parietal activity^35^.

Taken together, it can be hypothesized that verbal and figural fluency tasks depend on DLPFC activity and at the same time benefit from modality-specific brain areas and their respective cognitive functions. Yet, the extent to which they benefit from DLPFC activation and its executive resources is not well-studied. It is, therefore, crucial to investigate which brain region is involved to which degree in verbal and figural fluency and their related cognitive functions and whether these functions share similar or different neural processing. The answer to this question has also implications for a better understanding of DLPFC role in various EFs. Technological advances in cognitive neuroscience allow us to utilize a variety of techniques (e.g., neuroimaging, brain stimulation methods) to localize cognitive functions. Transcranial direct current stimulation (tDCS) is a noninvasive brain stimulation technique established as a simple, effective and safe method to modulate cortical excitability^36,37^ and cognitive functions^38,39^, including executive functions^40–43^ in both healthy humans and patients^44–46^. TDCS uses relatively weak electric currents involving the flow of electric current from a positive (an anode) to a negative site (a cathode) leading to an increase and decrease of cortical excitability respectively^47^. TDCS can thus show how modulation of cortical activity in different brain regions (e.g., prefrontal, temporal and parietal cortices) influences the performance of verbal and figural fluency tasks. This allows us to understand neural processes underlying verbal and figural fluency, including the identification of areas relevant for fluency in general, independent from modality, and areas which have task-specific relevance.

The present study, therefore, aims to investigate how modulation of cortical excitability in the left DLPFC (l-DLPFC), left temporal cortex and posterior parietal cortex (PPC), alters the performance of verbal and figural fluency tasks. Specifically, here we hypothesize that (1) the l-DLPFC is involved in EFs independent of modality and is thus significantly involved in verbal and figural fluency, (2) the temporal cortex has a modality-specific contribution to semantic fluency and (3) the PPC is specifically involved in visuomotor and visuo-spatial abilities and therefore modality-specific contributes to figural fluency. We thus expect that enhancing activity over the l-DLPFC with anodal tDCS enhances performance in both, verbal and figural fluency tasks whereas, enhancing activity of the left mesial temporal cortex and right PPC enhances performance in the semantic verbal fluency and figural fluency tasks respectively.

## Methods

### Participants

Fifteen healthy adult participants blind to the study hypothesis and stimulation took part in the experiment (7 males, Mean age 26.21 years, *SD* = 3.46). Demographic information of participants is shown in Table 1. The required sample size was calculated with G*Power 3.1, provided freely by the University of Duesseldorf^48^. The results of this power analysis showed that for running a repeated measures ANOVA with four measurements, a power of 0.95, an alpha level of 0.05, and a large effect size (f = 0.40), the required sample size is 15. The inclusion criteria were: (1) no previous history of brain surgery involving implants to the head, epilepsy, seizures, brain damage, head injury or loss of consciousness (2) no history of chronic or acute neurologic, psychiatric, or medical disease (3) no history of drug addiction and tobacco consumption and (4) no current pregnancy. The SCL-25 questionnaire^49^ that measures general psychological health was employed to initially screen participants’ psychological state before the beginning of the study. All participants were native speakers, right-handed and had normal or corrected-to-normal vision. Written informed consent was obtained from all participants and they were free to withdraw from the experiment at any stage. The experiment was approved by the ethical committee of the Shahid Beheshti University and the study was conducted in accordance with the latest version of the Declaration of Helsinki.

**Table 1:** Demographic information of participants.

Variable	Group	n
Sample size (*n*)		15
Gender	Male (Female)	7 (8)
Age	Mean (SD)	26.21 (3.46)
Education	Bachelor degree	8
	Masters degree	7
Marital status	Single (married)	11 (4)

### Materials

#### Verbal fluency task

We employed the verbal fluency task as a measure of verbal fluency. The task consisted of two parts: semantic (categorical) fluency and phonemic (letter) fluency test^50^. In the semantic verbal fluency task, subjects were asked to generate as many different words as possible belonging to each of the following categories: fruits, animals. Participants had 1 min for generating words in each semantic category. Semantic categories were selected based on the broadness of the considered semantic category (broad category for “animal” and intermediate category for “fruit”). Participants were instructed not to provide the same word twice (perseverative error) or produce nonwords. The first instruction (production of animal or fruit names) was given just before the beginning of data collection and the second instruction (to produce names of the other category, which means fruit names if animal names were required by the first instruction or vice versa) was given 1 min later. The order of categories was counterbalanced. During the phonemic verbal fluency task subjects were required to produce words beginning with a specific letter (the letters “F (ف)” and “J (ج)”) for 1 min each. Subjects were instructed not to provide the same word twice (perseverative error) or produce nonwords. Scores in this test are based on the number of generated words and the numbers of errors/repeated words. The words which did not meet the criteria (e.g., repeated words, nonwords) were classified as errors. The order of presentation of each of the two categories and each of the two letters was randomized and counterbalanced across participants. The order in which the phonemic and semantic verbal fluency tasks were administered was also counterbalanced across subjects. Subjects’ answers were tape-recorded and transcribed for analysis. The verbal fluency task, which was shown to have adequate reliability and validity^50^, was administered in the native language of the participants.

#### Five Point Test (FPT)

**Figure 1:**
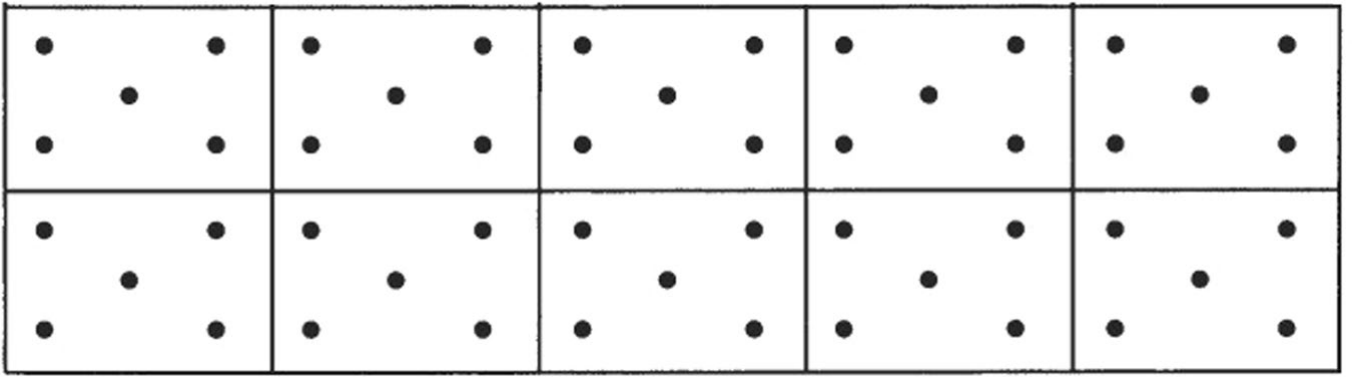
Test material for the five-point test. The test consists of a sheet of paper with 40 dot matrices arranged in eight rows and five columns as above. Participants are asked to produce as many different figures as possible by connecting the dots within each rectangle. *Note*: from Regards *et al*.^51^.

The FPT is a valid and reliable test of figural fluency^25^ developed by Regard *et al*.^51^. The standard application of the test procedure and the instructions for evaluation of test performance ensure a high level of objectivity^13^. We used the FPT because of its relative advantages over other figural fluency measures (e.g., RFFT) including brevity and simplicity. Studies report practice effects for RFFT, while such effects have not been found for all figural fluency tests^52^. Moreover, the RFFT uses five different dot matrices creating a potentially more complex task which requires figure-ground separation^53^. The FPT stimulus material consists of a page on which 40 identical squares are printed in eight rows and five columns, each square containing five symmetrically arranged dots (Fig. 1). Participants are asked to draw as many different figures as possible in 5 minutes by connecting two or more dots with straight lines. Participants were informed that not all the dots had to be used. Each participant was instructed not to repeat figures or draw lines which do not connect dots. At the start of the test, two sample solutions were drawn by the examiner. The test was administered using a standard oral instruction suggested by Regards *et al*.^51^. Scoring includes counting the total number of unique designs and the number of repeated designs (perseverative errors) drawn. Because the number of unique designs drawn by participants can influence the number of perseverative errors, the percentage of perseverative errors (i.e., perseverative errors + total unique figures × 100) was also calculated. The scoring procedure was done by a blinded rater.

### tDCS protocol

**Figure 2:**
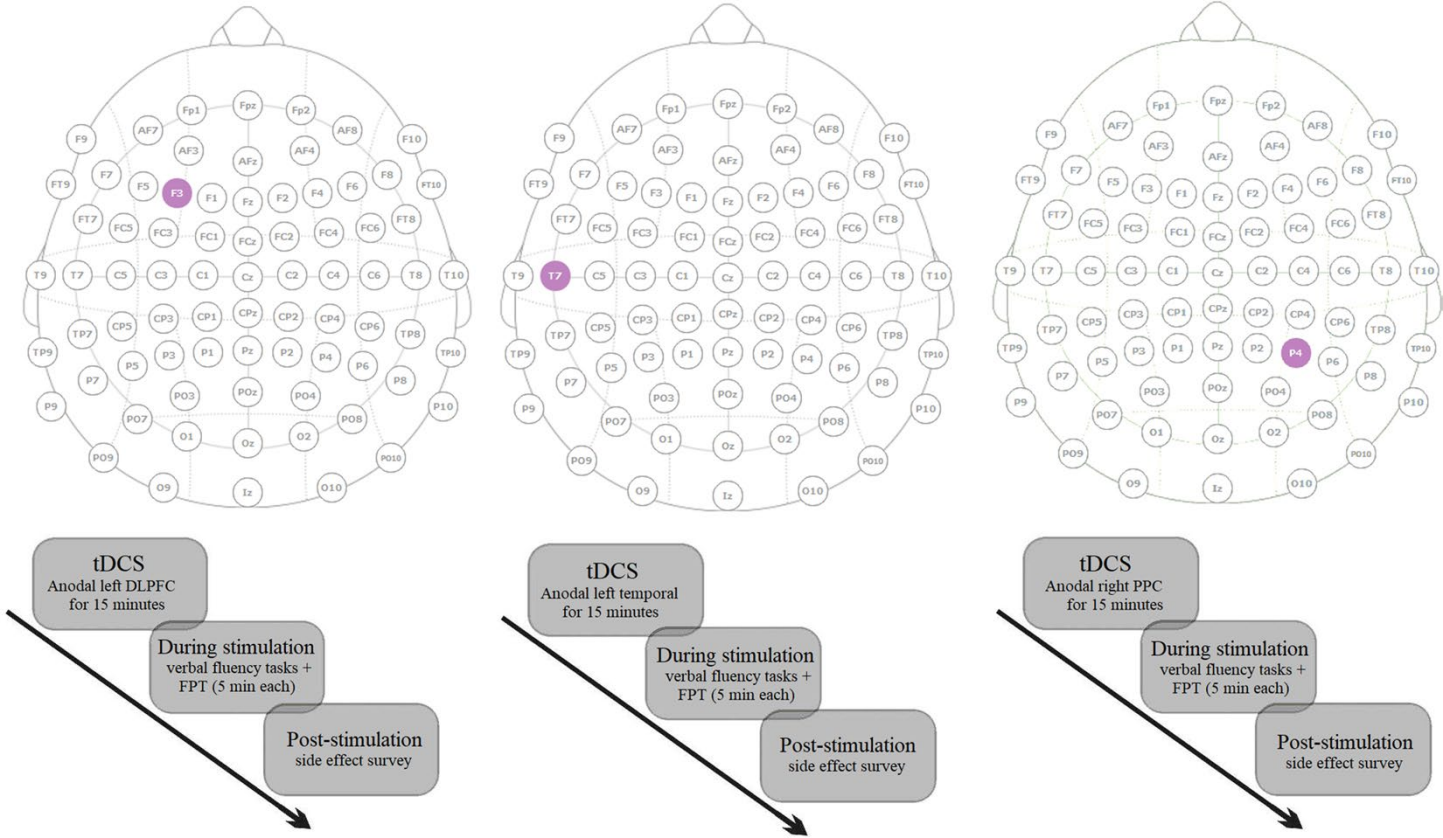
Experimental procedure regarding real tDCS conditions; tDCS = transcranial direct current stimulation; DLPFC = dorsolateral prefrontal cortex (F3); Left temporal = left temporal cortex (T7); PPC = Posterior parietal cortex (P4); FPT = Five point test. *Notes*: (**a**) F3 was used for sham tDCS too, (**b**) stimulation conditions and task order were counterbalanced across participants, (**c**) Electrode size used in this experiment was 7 × 5 cm, (**d**), T7 is analogous of T3 of 10–20 EEG system in the new high-resolution electrode-naming-system.

For brain stimulation, we used the “ActivaDose II Iontophoresis” Delivery Unit manufactured by Activa Tek, with a 9-volt battery as current source. Electrical direct current of 1.5 mA generated by the stimulator was applied through a pair of saline-soaked sponge electrodes with a size of 35 cm^2^ (7 × 5) for 15 min (with 15 s ramp up and 15 s ramp down). Four tDCS conditions were applied in this study: (a) anodal l-DLPFC tDCS, (b) anodal l-temporal tDCS, (c) anodal r-PPC tDCS, and (d) sham tDCS. The anodal electrode was positioned over F3 (left DLPFC), T3 (left temporal) and P4 (right PPC) according to the 10–20 EEG International System. The cathodal electrode was positioned over the contralateral shoulder. For the sham stimulation, anodal and cathodal electrodes were positioned over the F3 and contralateral shoulder respectively and electrical current was ramped up for 30 seconds to generate the same sensation as the active condition and then turned off without the participants’ knowledge^54^. This method of sham stimulation has been shown to be reliable^55^. All participants were blind to the type of stimulation they received. A side-effect survey was done after each tDCS session (Fig. 2).

### Procedure

Prior to the experiment, participants completed a brief questionnaire to evaluate their suitability for brain stimulation. All participants received 15 min of anodal l-DLPFC, anodal l-temporal, anodal r-PPC and sham stimulation with a 72-hr interval between each stimulation session in order to prevent transfer and confounding effects of stimulation. After five minutes of stimulation during which participants sat at rest, they performed the verbal fluency and the FPT tasks while they received electrical brain stimulation (Fig. 2). The verbal tasks were performed in the native language of the participants. The order of the task and polarity of stimulation were counterbalanced across participants. Participants were orally instructed about each task before the beginning of the experiment. Each task required around 5 minutes to complete making a total of 10 minutes required for completing both tasks. The location of stimulation (i.e., DLPFC, temporal cortex, PPC) was also randomized across participants in order to control for “order effects”. The experiment was conducted in the Cognitive Neuroscience laboratory at the Department of Psychology, Shahid Beheshti University.

### Statistical analysis

This study had a double-blind, within-subjects, single factor design. The experimenter was blind to stimulation polarities and participants were blind to both stimulation polarities and study hypotheses. Data analyses were conducted using the statistical package SPSS for Windows, version 24 (IBM, SPSS, Inc., Chicago, IL). Normality and homogeneity of variance of data collected from each stimulation condition were confirmed using the Shapiro-Wilk and Levin tests respectively. To estimate the effect of tDCS on performance of the fluency tasks, a two-factor repeated measures analysis of variance (ANOVA) was carried out on the dependent variables (i.e., the number of generated words in semantic and phonemic fluency tests and the number of unique designs and perseverative errors in FPT) with tDCS conditions (i.e., anodal l-DLPFC, anodal r-PPC, anodal left temporal, sham) and task condition (i.e., semantic fluency, phonemic fluency, figural fluency) as within-subject factors. Mauchly’s test was used to evaluate the sphericity of the data before performing the repeated measures ANOVA for each dependent variable. Post hoc analysis was carried out using Bonferroni-corrected post hoc *Student´s* t-tests. Mauchly’s test of sphericity showed that sphericity was not violated and thus no correction of the degrees of freedom was required. We also added an analysis of covariance (ANCOVA) with the order of stimulation as a covariate to control for its potential confounding effect. For calculating blinding success in participants, their guess of stimulation quality (i.e., active vs sham stimulation) was compared using a chi-square test. Fisher’s exact test was also used for comparing reported side effects after each session. A significance level of *p* < 0.05 was used for all statistical comparisons.

## Results

### Data overview

**Figure 3:**
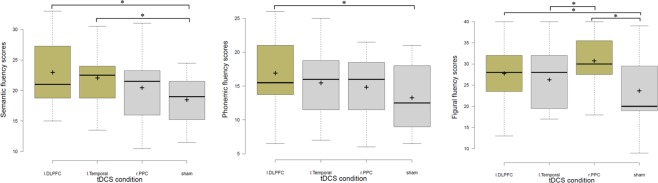
Performance of the semantic (average scores of “fruit” and “animal” categories), phonemic (average scores of “letter F” and “letter J” categories) and figural fluency tasks during tDCS. Significant conditions are coloured. *Note*: tDCS = transcranial direct current stimulation; l-DLPFC = left dorsolateral prefrontal cortex; l-temporal = left temporal cortex; r-PPC = right posterior parietal cortex; * = indicates significant pairwise comparisons following Bonferroni post-hoc correction at adjusted *p*-value; Error bars indicate 95% confidence intervals; Boxes indicate interquartile range that contains 50% of values (range from the 25th to the 75th percentile).

All participants tolerated tDCS well and no adverse effects were reported during and after stimulation except for a mild itching, tingling and burning sensation under the electrodes during approximately the first 30 s of stimulation in each tDCS condition. The occurrence of side effects is summarized in Table 2. Moreover, the perceived side effects and evaluations after each tDCS session were not significantly different between stimulation conditions. The data overview shows that performance of verbal fluency and FPT tasks differed between tDCS conditions (Fig. 3). The mean and standard deviation of task performance parameters are presented in Table 3. Demographic variables were not significantly correlated with the dependent variables and thus were not included as covariates in the analysis. Analyses were performed for the mean number of words generated excluding errors in the phonemic (averaged number of words for each letter) and semantic fluency tasks (averaged number of words for each category) and the number of unique figures generated as well as number/percentage of errors (as secondary analysis) in the FPT. For blinding of stimulation, the respective results showed that the proportions of participants who thought they had received active stimulation, sham stimulation or who did not know, did not significantly differ between real and sham stimulation conditions (χ^2^ = 5.11, p = 0.27), which is in accordance with an efficient blinding procedure.

**Table 2:** Reported tDCS side effects during stimulation.

tDCS session	Tingling	Itching sensation	Burning sensation	Pain	Fatigue	Trouble concentrating
Anodal l-DLPFC	14 (2.7)	13 (2.4)	11 (2.3)	3 (2)	2 (1.7)	0
Anodal l-temporal	13 (2.7)	11 (2.5)	12 (2)	2 (2)	1 (2)	1 (1)
Anodal r-PPC	14 (2.6)	12 (2.6)	11 (2.4)	2 (2)	2 (1.9)	1 (2)
Sham	11 (2.4)	10 (2.2)	10 (2)	1 (1)	1 (2)	1 (1)
*χ* ^2^_*(active vs sham)*_	0.39	1.87	0.68	0.26	0.16	0.08
*P*	0.73	0.24	0.56	0.80	0.86	0.93

Numbers in the parentheses indcate mean of intensity of reported side effect. tDCS = transcranial direct current stimulation; l-DLPFC = left dorsolateral prefrontal cortex; l-temporal = left temporal cortex; r-PPC = right posterior parietal cortex.

**Table 3:** Means and *SD*s of the verbal fluency task and Five Point Test performance under different tDCS conditions.

Task	Dependent variables	tDCS condition			
		Anodal l-DLPFC tDCS	Anodal l-temporal tDCS	Anodal r-PPC tDCS	Sham tDCS
		*M* (*SD*)	*M* (*SD*)	*M* (*SD*)	*M* (*SD*)
Verbal fluency	Semantic fluency*	22.80 (5.31)	21.90 (4.40)	20.30 (5.41)	18.33 (3.97)
Verbal fluency	Phonemic fluency**	16.76 (5.29)	15.33 (5.51)	14.70 (5.04)	13.13 (4.84)
Figural fluency	Uniqe figures	27.53 (7.45)	26.06 (7.01)	30.53 (6.47)	23.46 (8.15)
	Perseverative errors	3.4 (3.39)	2.6 (1.63)	2.66 (3.22)	2.93 (2.05)
	% of perseverative errors	13.87 (14.57)	10.65 (6.7)	9.77 (12.21)	12.85 (9.02)

*Note*: tDCS = transcranial direct current stimulation; l-DLPFC = left dorsolateral prefrontal cortex; l-temporal = left temporal cortex; r-PPC = right posterior parietal cortex; M = Mean; SD = Standard Deviation; * = Mean number of words produced during semantic fluency task (Represents pooled data from “fruit” and “animal” subcategories); ** = Mean number of words produced during phonemic fluency task (Represents pooled data from Letter “F” and Letter “J” subcategories). Respective statistics for each subcategory can be found in supplementary notes.

### Differential effects of tDCS on fluency performance

In order to investigate effects of tDCS on fluency tasks performance, we conducted a two-factor repeated measures ANOVA) with stimulation protocol (i.e., anodal l-DLPFC, anodal left temporal, anodal r-PPC, sham) and task condition (i.e., figural fluency, semantic fluency and phonemic fluency) as within-subject factors. The results show a significant interaction effect of stimulation protocol and task condition (*F*_*(6,84)*_ = 7.48, *p* < 0.001, *η*_**p**_^2^ = 0.35) indicating that effects of stimulation depend on task modality (i.e., semantic, phonemic, figural) which confirms differential effects of stimulation on task performance. In addition, the main effect of stimulation protocol was significant (*F*_(3,42)_ = 27.72, *p* < 0.001, *η*_**p**_^2^ = 0.66) indicating that tDCS significantly improved performance on the fluency tasks. Similarly, a significant main effect of task condition was also observed (*F*_(2,28)_ = 64.50, *p* < 0.001, *η*_**p**_^2^ = 0.82) showing that task modality affects performance. Results are displayed in Table 4.

**Table 4:** Results of the two-factor repeated measures ANOVA for effects of tDCS conditions (anodal l-DLPFC/anodal l-temporal/anodal r-PPC/sham) and task conditions (semantic, phonemic, figural) on verbal and figural fluency tasks.

Task	*df*	Mean square	*F*	*p*	*η*_*p*_^*2*^
tDCS conditions	3, 42	146.78	27.72	**<0.001**	0.66
Task conditions	2, 28	2130.33	64.50	**<0.001**	0.82
tDCS*task	6, 84	37.68	7.48	**<0.001**	0.35

*Note:* tDCS = transcranial direct current stimulation; l-DLPFC = left dorsolateral prefrontal cortex; l-temporal = left temporal cortex; r-PPC = right posterior parietal cortex; *η*_***p***_^*2*^ = partial eta squared; Significant results are highlighted (*p* ≤ 0.05) in bold.

The differential effects of tDCS on task performance were further investigated by post-hoc tests. The Bonferroni-corrected post hoc analysis showed that participants generated significantly more words, indicative of semantic fluency, only during real tDCS over the l-DLPFC (*t* = 4.46, *p* < 0.001; *M* = 22.80, *SD* = 5.31) and left temporal cortex (*t* = 3.56, *p* < 0.001; *M* = 21.90, *SD* = 4.40), but not during anodal r-PPC tDCS (*t* = 1.96, *p* = 0.555, *M* = 20.30, *SD* = 5.41), as compared to sham (*M* = 18.33, *SD* = 3.97). This was true for both semantic categories (i.e., “fruit” and “animal”) and indicates that semantic fluency, regardless of semantic category, improved as a result of increased activation of the l-DLPFC and left temporal cortex. Sub-analyses of the results of each subcategory can be found in the supplementary material.

Regarding phonemic fluency, the results of the Bonferroni-corrected post hoc analysis indicate that participants significantly produced more words, regardless of the phonemic cue (i.e., letter “F” and letter “J”), only during anodal tDCS over the l-DLPFC compared to sham tDCS (*t* = 3.63, *p* < 0.001; *M* = 16.76, *SD* = 5.29 vs. *M*_*sham*_ = 13.13, *SD*_*sham*_ = 4.84) but not during anodal l-temporal tDCS (*t* = 1.43, *p* = 0.704; *M* = 15.33, *SD* = 5.51), or anodal r-PPC tDCS (*t* = 2.06, *p* = 0.060; *M* = 14.70, *SD* = 5.04). This was true for both phonemic cues and indicates that phonemic fluency significantly improved as a result of increased activation of the l-DLPFC only. Sub-analyses of each phonemic subcategory can be found in the supplementary material.

Finally, with regard to the figural fluency performance, the Bonferroni-corrected post hoc tests show that participants produced significantly more unique and novel figures under anodal r-PPC tDCS (*t* = 7.06, *p* < 0.001; *M* = 30.53, *SD* = 6.47) and anodal l-DLPFC tDCS (*t* = 4.06, *p* < 0.002; *M* = 27.53, *SD* = 7.45), but not under anodal l-temporal tDCS (*t* = 2.60. *p* = 0.405; *M* = 26.06, *SD* = 7.01), as compared to sham tDCS (*M* = 23.46, *SD* = 8.15). Furthermore, although both, anodal r-PPC and anodal l-DLPFC improved figural fluency, the former montage produced a larger effect size and the difference between the two montages (*M* = 30.53 vs. *M* = 27.53) was close to significance (*p* = 0.073), implicating that anodal r-PPC tDCS had a stronger effect on figural fluency than anodal l-DLPFC tDCS. The ANCOVA results show that the order of stimulation did not have a significant effect on the dependent variable (*F*_*(1,55)*_ = 0.12, *p* = 0.73). The effect of tDCS condition on the number and percentage of perseverative errors (*F*_*(3,4*2*)*_ = 0.31, *p* = 0.81, *η*_**p**_^2^ = 0.02) and the percentage of perseverative errors (*F*_*(3,42)*_ = 0.53, *p* = 0.65, *η*_**p**_^*2*^ = 0.04) were also investigated and the ANOVA results show no significant effect of tDCS on the number of errors, although the percentage of error rate was numerically lower under anodal r-PPC tDCS (See Table 3).

## Discussion

The present study investigated the effects of increasing cortical excitability of the l-DLPFC, left temporal cortex and r-PPC with anodal tDCS on verbal and figural fluency. We found that anodal stimulation of the l-DLPFC improved performance on fluency tasks independent of modality (i.e., semantic fluency, phonemic fluency, figural fluency) supporting the first hypothesis, while anodal stimulation of temporal and posterior parietal cortices had modality-specific enhancing effects on semantic and figural fluency respectively, which supports the second and third hypotheses. Although several studies investigated effects of tDCS on verbal fluency^56–58^, to our knowledge this is the first study to investigate differential causal roles of the prefrontal, temporal and parietal cortices in verbal and figural (non-verbal) fluency at the same time.

The results of the study suggest that all modalities of fluency (i.e., semantic, phonemic, figural) are significantly associated with left prefrontal activation and performance of the respective tasks improved as a result of left DLPFC activation. The l-DLPFC is a prefrontal region primarily responsible for EFs or cognitive control^28,42^ and its significant role in fluency, found in this study, implicates that EFs might be important in fluency independent of specific modalities too. In other words, l-DLPFC activation has supramodal effects on semantic, phonemic and figural fluency possibly through the supramodal contribution of l-DLPFC-related executive resources. This is in accordance with previous studies that established a close relationship between DLPFC-supported EFs and different fluency modalities^4,8–10^. Phonetic fluency involves strategic search and retrieval processes within orthographic or phonological networks^59^ and thus requires a number of higher order EFs including working memory, inhibition, switching, and set-shifting^11^. Similarly, semantic fluency reflects both, executive and associative retrieval mechanisms particularly reliant upon intact semantic networks and depends on higher order executive resources of the DLPFC^11,60^. Figural fluency depends on selective attention, maintaining visual information and inhibiting repetition of design, which also is dependent on DLPFC executive resources. In accordance, findings from clinical populations confirm that patients with prefrontal and frontal lesions have impaired performance on all fluency tasks^22–24,30^. In further accordance, imaging and brain stimulation studies show greater activation in the left hemisphere during category and letter fluency tasks^19^ and enhanced EFs and fluency after anodal stimulation of left prefrontal regions^56,58,61,62^.

Based on the results of the present study, we cannot however conclude with certainty that such supramodal effects of l-DLPFC activation on different fluency task are transmitted only via enhanced EFs. In other words, although executive resources are dependent on l-DLPFC activation, we cannot claim that EFs, as an independent variable, affected fluency performance. This is, however, a reasonable speculation which should be confirmed in future studies by actively manipulating EFs components and examining its effects of fluency tasks.

In addition to supramodal contribution of the l-DLPFC activation, we also found that performance in each fluency modality depends on activation of modality-specific brain regions, namely the temporal cortex for semantic fluency and PPC for figural fluency. Our results showed that semantic fluency significantly improved after activation of the left temporal cortex, implicating that the left temporal region is specifically relevant for this function. This indicates that this temporal area has a modality-specific contribution to semantic fluency. Involvement of the temporal cortex in semantic knowledge and semantic word fluency is well documented in previous studies in healthy and neurological populations^63–66^. Moreover, recent evidence suggests a pattern of distinct and shared correlates of semantic and phonemic fluency at the anatomical and cognitive level^6^. The left medial temporal region seems to be specifically involved in semantic fluency, whereas the left prefrontal cortex has been suggested to be relevant for both, semantic and phonemic tasks^5^. Similarly, a distinct set of cognitive processes, specifically with regard to search strategies and recruitment of working memory is suggested to underlie semantic and phonemic fluency^6^. These findings are in line with our results which show that semantic and phonemic fluency both benefit from l-DLPFC activation, while left temporal activation exclusively enhanced semantic fluency.

Similarly, in addition to the significant contribution of the l-DLPFC in figural fluency, we found a modality-specific contribution of the r-PPC in figural fluency, which was exclusively relevant for this function. Involvement of the r-PPC in figural fluency is in line with results of previous studies indicating the importance of the r-PPC for cognitive functions required for performance on figural fluency tests including visuospatial attention^16,67^, visual memory retrieval^17,68^ spatial representation/updating^69^, visually-guided motor planning^33^ and retrospective coding of visual space^16^, which are all important for performance of a task like the FPT. This result also replicates our recent results about the enhancement of figural fluency after anodal-PPC tDCS^70^. One might ask why other regions in the right hemisphere, including the r-DLPFC, were not stimulated during the figural fluency task. We targeted the PPC rather than DLPFC in the right hemisphere because the r-PPC is a more relevant region for figural fluency involving the visuo-spatial modality, while the DLPFC is usually targeted more in studies using fluency/cognitive flexibility tasks with verbal modalities^71–73^. Secondly, adding another stimulation condition in our study would have increased the risk for ceiling effects and task repletion relevantly. However, the r-DLPFC is still a potentially interesting region to study in future tDCS studies on figural fluency using unilateral (i.e., r-DLPFC tDCS) or bilateral stimulation montages (i.e., left and right DLPFC tDCS).

Taken together, our findings suggest that the l-DLPFC has a significant supramodal contribution to fluency and executive functioning. Additionally, a modality-specific pattern was observed for the involvement of the temporal and parietal cortices in semantic and figural fluency. The concept of modality specificity is well-documented for semantic memory, however, our results indicate a similar concept for figural fluency too. These results have at least the following implications regarding the hypothesized fluency-specific contribution of EFs: first of all, it suggests a higher-order supervisory role of DLPFC activation in exerting control over multimodal sensory information which is relevant for various cognitive functions in favor of goal-directed actions. This is in line with the non-modular contribution of the PFC regions, including the DLPFC, in cognitive functions^42,74^. However, we need to distinguish non-modular contributions of l-DLPFC activation from a specific impact of EFs which were not directly manipulated in the current study. In order to come to clear assumptions about the supramodal contribution of EFs to fluency, it is required to associate the effects of DLPFC-related EF components (e.g., attentional control, working memory, inhibitory control), more directly with verbal and figural fluency tasks. Secondly, the concept of “functional specificity” or “modularity” is supported by our results regarding semantic and figural fluency, where additionally stimulation of respective temporal and parietal cortices improved performance, thus adding information about the causal relevance of these areas for respective cognitive functions. Given the feature of tDCS to explore the causal role of the respective areas for performance via modulation of respective cortical activity, these results add important information to those of neuroimaging studies. Nonetheless, further evidence from combined stimulation-neuroimaging studies (i.e., EEG, fMRI) would be helpful to further support this concept. Additional to “functional specificity” implication, results of our study also show the specificity of tDCS effects and might imply advantages of network stimulation approaches for improving functions in future studies.

Despite promising results, some limitations of our approach should be taken into account: The low spatial resolution of tDCS is an inherent limitation of this noninvasive brain stimulation technique, which should be considered. Therefore, the involvement of adjacent areas in stimulation effects cannot be ruled out completely. Potential practice effect could have furthermore been present for the figural fluency task. Although the FPT is not usually affected by practice effects as much as other tasks, such as the RIFF^52^ and despite the randomized presentation of tasks during each stimulation condition, it is still possible that FPT performance is affected by practice effects. The same aspects should be considered for interpreting the results of the verbal fluency task, since participants were allowed to generate the same words across different stimulation conditions, which might have made task performance easier in the later conditions. Although stimulation conditions were randomized across participants and an ANCOVA was conducted rule out order effects, the results still need to be interpreted cautiously and future studies are required to resolve the impact of this potentially confounding variable. Moreover, adding some control tasks that monitor potential confounding variables (i.e., motor speed) would have allowed to draw more robust conclusions about the cognitive effects of tDCS in this study. With regard to the tolerability of tDCS, results of the side effect survey showed no adverse side effects during and following stimulation. It might be advantageous to examine tDCS side effects in a pre-post measure design in future studies. Lastly, due to the relatively small sample size, the results of this study are preliminary and should be confirmed by larger subsequent studies.

In conclusion, fluency, as one of the major EF domains, is dependent on the global contribution of DLPFC activation and at the same time specifically relies on respective modality-specific brain areas and cognitive functions. A similar pattern might be true for other components of EFs (i.e., inhibition, updating, task switching) which need to be examined in future studies. Finally, it is noteworthy that our study implies potential enhancing effects of tDCS on verbal and figural fluency which might suggest a potential for therapeutic use of tDCS in disorders involving language impairment and impaired nonverbal fluency such as in brain injury, dementia, aphasia or stroke.

## Supplementary information

Supplementary results

## Data Availability

The datasets generated during and/or analyzed during the current study are available from the corresponding author on reasonable request.
