# Supplementary material for: Differential role of prefrontal, temporal and parietal cortices in verbal and figural fluency: Implications for the supramodal contribution of executive functions
Source: Sci Rep. 2019 Mar 6;9:3700. doi: 10.1038/s41598-019-40273-7 (PMC6403289; doi:10.1038/s41598-019-40273-7)
Supplement: Supplementary file 1 — Supplementary results [file 41598_2019_40273_MOESM1_ESM.pdf]

## Title

Differential role of prefrontal, temporal and parietal cortices in verbal and figural fluency: Implications for the supramodal contribution of executive functions

## Authors

Elham Ghanavati[+1,2], Mohammad Ali Salehinejad*[+ 2,3,4], Vahid Nejati*[5,6], Michael A. Nitsche[2,7]

[1] Department of Psychology, Islamic Azad University, Science & Research Branch, Tehran, Iran
[2] Leibniz Research Centre for Working Environment and Human Factors, Department of Psychology and Neurosciences, Dortmund, Germany
[3] Institute for Cognitive and Brain Sciences, Shahid Beheshti University, Tehran Iran
[4] Ruhr University Bochum, International Graduate School of Neuroscience, Bochum, German
[5] Faculty of Psychology and Educational Sciences, Department of Psychology, Shahid Beheshti University, Tehran, Iran
[6] University of Regensburg, Department of Psychology, Regensburg, Germany
[7] University Medical Hospital Bergmannsheil, Department of Neurology, Bochum, Germany

*Corresponding Authors:
M. A. Salehinejad: salehinejad@ifado.de / salehinejadmohammadali@gmail.com
V. Nejati: nejati@sbu.ac.ir
[+]These authors contributed equally

## 1. Results (supplementary)

*Differential effects of tDCS on semantic and phonemic fluency subcategories*

The differential effects of tDCS on each subcategory of task performance were further investigated using the post-hoc tests. The Bonferroni-corrected post hoc analysis showed that participants generated significantly more words in the fruit category only during real tDCS over the l-DLPFC ($t$ = 3.61, $p$ < 0.01; $M$ = 19.20, $SD$ = 5.17) and left temporal cortex ($t$ = 3.13, $p$ < 0.02; $M$ = 18.73, $SD$ = 4.39), but not during anodal r-PPC tDCS ($t$ = 1.13, $p$ = 0.58, $M$ = 16.73, $SD$ = 3.75), as compared to sham ($M$ = 15.6, $SD$ = 4.62). Similarly, the Bonferroni-corrected post hoc analysis showed that participants generated significantly more words in the animal category only during anodal l-DLPFC tDCS ($t$ = 5.33, $p$ < 0.01; $M$ = 26.4, $SD$ = 6.15) and anodal l-temporal tDCS ($t$ = 4, $p$ < 0.01; $M$ = 25.06, $SD$ = 5.58) but not during anodal r-PPC tDCS ($t$ = 2.80, $p$ = 0.22; $M$ = 23.86, $SD$ = 6.96) compared to sham tDCS ($M$ = 21.06, $SD$ = 4.04).

Regarding phonemic fluency, the results of the Bonferroni-corrected post hoc analysis indicate that participants significantly produced more words beginning with the letter "F" only during anodal tDCS over the l-DLPFC compared to sham tDCS ($t$ = 3.26, $p$ < 0.01; $M$ = 16.13, $SD$ = 5.13 vs. $M_{sham}$ = 12.86, $SD_{sham}$ = 5.56) but not during anodal l-temporal tDCS ($t$ = 2.86, $p$ = 0.07; $M$ = 15.73, $SD$ = 5.59), or anodal r-PPC tDCS ($t$ = 2.13, $p$ = 0.34; $M$ = 15, $SD$ = 5.66). The Bonferroni-corrected post hoc analysis similarly indicates that participants produced significantly more words beginning with the letter "J" only during anodal l-DLPFC tDCS compared to sham tDCS ($t$ = 4.02, $p$ < 0.01; $M$ = 17.04, $SD$ = 5.67 vs. $M_{sham}$ = 13.40, $SD$ = 4.35) but not during anodal l-temporal tDCS ($t$ = 1.53, $p$ = 0.07; $M$ = 14.93, $SD$ = 5.21) or anodal r-PPC tDCS ($t$ = 1, $p$ = 0.71; $M$ = 13.40, $SD$ = 4.35). The

ANCOVA results ($F_{fruit}$ = 0.68, $p$ = 0.79; $F_{animal}$ = 0.93, $p$ = 0.76; $F_{letter"F"}$ = 0.01, $p$ = 0.95; $F_{letter"J"}$ = 0.02, $p$ = 0.89) demonstrated no significant effect of stimulation order on semantic and phonemic fluency performance for each subcategory.

**Table 3.** Means and *SD*s of the semantic and phonemic fluency task performance under different tDCS conditions.

| | | **tDCS condition** | | | |
|---|---|---|---|---|---|
| | | **Anodal l-DLPFC tDCS** | **Anodal l-temporal tDCS** | **Anodal r-PPC tDCS** | **Sham tDCS** |
| **Task** | **Dependent variables** | ***M* (*SD*)** | ***M* (*SD*)** | ***M* (*SD*)** | ***M* (*SD*)** |
| **Verbal fluency** | Semantic fluency (overall) | 22.80 (5.31) | 21.90 (4.40) | 20.30 (5.41) | 18.33 (3.97) |
| | *"Fruit" | 19.20 (5.17) | 18.73 (4.39) | 16.73 (3.75) | 15.6 (4.62) |
| | *"Animal" | 26.4 (6.15) | 25.06 (5.58) | 23.86 (6.96) | 21.06 (4.04) |
| **Verbal fluency** | Phonemic fluency (overall) | 16.76 (5.29) | 15.33 (5.51) | 14.70 (5.04) | 13.13 (4.84) |
| | *Letter "F" | 16.13 (5.13) | 15.73 (5.59) | 15 (5.66) | 12.86 (5.56) |
| | *Letter "J" | 17.4 (5.67) | 14.93 (5.21) | 14.4 (5.71) | 13.40 (4.35) |

tDCS = transcranial direct current stimulation; l-DLPFC = left dorsolateral prefrontal cortex; l-temporal = left temporal cortex; r-PPC = right posterior parietal cortex; M = Mean; SD = Standard Deviation; * = Mean number of words produced for respective category.
